# Leveraging Public Health Informatics Through the Data–Information–Knowledge–Wisdom (DIKW) Framework in Community-Based Surveillance of Bangladesh

**DOI:** 10.3390/tropicalmed11070181

**Published:** 2026-06-29

**Authors:** Immamul Muntasir, Md. Omar Qayum, Arifa Hasnat Ali, Fahim Mohammad Sadique Srijon, Mohammad Rashedul Hassan, Mahbubur Rahman, Tahmina Shirin

**Affiliations:** 1 Institute of Epidemiology, Disease Control and Research (IEDCR), Mohakhali, Dhaka 1212, Bangladesh; oqayum84@gmail.com (M.O.Q.); mrh.russel@gmail.com (M.R.H.); dr_mahbub@yahoo.com (M.R.); tahmina.shirin14@gmail.com (T.S.); 2 Danish Red Cross, International Federation of Red Cross and Red Crescent Societies, Dhaka 1217, Bangladesh; arali@rodekors.dk; 3 Bangladesh Red Crescent Society (BDRCS), Dhaka 1217, Bangladesh; fahim.srijon@bdrcs.org

**Keywords:** community-based surveillance, public health informatics, early outbreak detection, suspected dengue, influenza-like illness, Bangladesh, DIKW

## Abstract

Early detection of infectious disease outbreaks is critical in densely populated, resource-limited settings. This study aimed to describe the community-based surveillance (CBS) system and its application of the Data–Information–Knowledge–Wisdom (DIKW) framework in Bangladesh. CBS was implemented in 12 urban wards across Dhaka South, Rajshahi, and Sylhet, where trained community volunteers conducted routine household visits to identify five priority syndromes. Data were collected through a mobile application integrated with an automated pipeline for cleaning, geocoding, cluster detection, and alert generation. Between January and June 2025, 38,489 households were visited, enrolling 128,626 individuals. The system generated 10,191 alerts and 577 clusters, predominantly for suspected dengue (58.7%), followed by acute watery diarrhea (24.1%) and influenza-like illness (10.7%). Rajshahi contributed the majority of alerts and clusters. Spatiotemporal analysis identified ward-level outbreak signals, including localized dengue peaks across all three cities. Over 98% of records were synchronized within 24 h, and more than 99% of data entry errors were automatically corrected, ensuring timely and high-quality analytics. These findings demonstrate that digital CBS can effectively transform community-level data into actionable public health intelligence, supporting early outbreak detection and response. This translation enabled timely public health actions, including targeted outbreak investigations and localized vector control measures in identified hotspots. Integration with national surveillance platforms may further strengthen health system responsiveness and epidemic preparedness.

## 1. Introduction

Public health informatics involves using information technology and data science to turn health data into actionable intelligence and improved health outcomes. In the classic Data–Information–Knowledge–Wisdom (DIKW) framework, raw signals (“data”) are processed into structured information, analyzed into knowledge about trends, and applied as “wisdom” in decision-making [1]. Community-based surveillance (CBS) is a public health approach that exemplifies this process: community volunteers collect raw symptom data, which are then digitally aggregated, cleaned, and analyzed to detect outbreaks. The World Health Organization (WHO) and related guidelines emphasize community participation in surveillance, defining CBS as “trained surveillance informants [community volunteers (CV)] identify and report events in the community that have public health significance” [2,3]. By decentralizing data collection, CBS provides near real-time community-level input that complements facility-based reporting [4].

Globally, CBS has proven valuable in low-resource and crisis settings. A scoping review identified 79 CBS systems across 42 countries (mostly low/middle income) [4]. These systems often target outbreak-prone diseases in rural or underserved areas that facility-based surveillance might miss [4,5]. For example, CBS was successfully used in campaigns against polio and cholera in Africa and for Zika and dengue in Latin America by enabling local event reporting before large outbreaks occurred [4,6,7,8]. In Indonesia, CBS increased case detection of suspected avian influenza, enhancing the understanding of its spread [9]. Similarly, CBS pilots in Ghana [10] (using GIS-linked mobile reporting) and Sierra Leone [11] (post-Ebola) demonstrated that communities can flag outbreaks early. In Ghana, intensifying CBS with geospatial mapping identified additional COVID-19 cases and was associated with a four-fold weekly increase in reported cases. In Sierra Leone, CBS alerts for acute diarrhea and other illnesses closely mirrored—and even added to—the national Integrated Disease Surveillance and Response (IDSR) reports, suggesting cases were caught that facilities would have missed [11].

Bangladesh is highly vulnerable to infectious disease outbreaks due to its dense population, frequent natural disasters, and gaps in surveillance system coverage [12]. For instance, a nationwide cholera surveillance analysis based on 22 sentinel hospital sites and the icddr,b Dhaka hospital found that approximately 69% of Bangladesh’s population lives outside the catchment areas of laboratory-confirmed cholera surveillance (“surveillance greyspots”) [5]. The coastal and flood-prone regions face recurrent outbreaks [13], and urban slums in Bangladesh lack robust early warning and surveillance systems, as residents face significant barriers to accessing formal healthcare and disease detection services, leading to delayed outbreak identification and response [14]. In the absence of routine CBS, outbreaks can spread undetected for days. Recognizing this, the Bangladesh Red Crescent Society (BDRCS), in partnership with the Institute of Epidemiology, Disease Control and Research (IEDCR), launched a CBS pilot in 2021 covering 12 wards across three city corporations (Dhaka South, Rajshahi, and Sylhet) [15,16] under the Epidemic and Pandemic Preparedness and Response (EPPR) pillar of the Pilot Programmatic Partnership (PPP), with the German Red Cross, Danish Red Cross, Swedish Red Cross and International Federation of Red Cross and Red Crescent Societies (IFRC) providing technical and financial support from the Humanitarian Aid Department of the European Commission (ECHO). This pilot used an open-source mobile app for CVs to collect symptom reports and was explicitly designed as a public health informatics intervention, with the aim of translating community-based data into timely surveillance intelligence.

In this paper, we describe the CBS pilot’s design and results, highlighting how informatics processes were operationalized. These include mobile data capture with real-time syncing, automated data cleaning and deduplication, algorithmic cluster detection with automated alerts, integration of data into analytical dashboards, and plans for linking with national surveillance platforms. We frame our findings within the DIKW model and compare them to international CBS experiences. We emphasize published evidence from Ghana, Indonesia, Sierra Leone, Niger and others to contextualize the Bangladesh case. Overall, we demonstrate that digitized CBS—when guided by informatics principles—can effectively transform community reports into actionable surveillance signals, enhancing outbreak detection in low-resource settings.

## 2. Materials and Methods

### 2.1. Surveillance System Design and Period

The Community-Based Surveillance (CBS) was designed as an observational study to establish an effective early detection and community reporting system for epidemic-prone diseases. The pilot phase continued from January to June 2025. The methodological framework is as follows:

- Feasibility assessment
- Site selection
- Development and validation of community case definitions
- Digital tool customization
- Volunteer recruitment and training
- Surveillance implementation, data processing, analysis, interpretation and data-driven action

### 2.2. Surveillance Sites and Population

The pilot was implemented in 12 purposively selected urban wards across three major city corporations of Bangladesh:

• Dhaka South City Corporation (DSCC)—2 wards
• Rajshahi City Corporation (RCC)—5 wards
• Sylhet City Corporation (SCC)—5 wards

Wards were selected based on high population density, vulnerability to climate-sensitive diseases, historical outbreak patterns, and operational feasibility. All residents within the selected wards were included in the surveillance population.

### 2.3. Recruitment and Training of Community Volunteers

Each ward was staffed by 20 community volunteers (CVs), all of whom were local residents, familiar with community geography and social dynamics. To enhance consistency across volunteers, standardized training and supervision were implemented. Training was provided jointly by BDRCS and IEDCR and covered:

• Recognition of five priority syndromes using community-adapted case definitions
• Use of the mobile CBS application
• Standards for household engagement and confidentiality
• Safe field practices and reporting protocols
• Procedures for escalation of unusual events or suspected clusters

Supervisors provided daily oversight, ensured proper syncing of mobile data, and maintained communication between CVs and health authorities.

### 2.4. Community Case Definitions

To ensure consistent reporting, simplified community-level case definitions were developed following WHO/IFRC CBS standards and validated by IEDCR. These were embedded in the CBS mobile app. Definitions covered:

• Suspected Dengue
• Influenza-like Illness (ILI)
• Acute Watery Diarrhea (AWD)
• Suspected Acute Jaundice Syndrome (AJS)
• Unusual Health Events (Human/Animal), defined as three or more people suddenly falling ill or dying with similar symptoms within a 1 km radius or 10 min walking distance over two weeks

These definitions enabled volunteers to identify cases during routine visits with minimal ambiguity (Table 1).

### 2.5. Cluster Detection

Cluster detection was based on the CBS definition of three or more suspected cases of the same syndrome within 500 m over a 14-day rolling window. This approach was informed by International Federation of Red Cross and Red Crescent Societies (IFRC) community-based surveillance guidelines, which emphasize the identification of “unusual health events,” including clusters of similar illnesses occurring within a defined area and time period at the community level. In line with these guidelines, which do not prescribe fixed quantitative thresholds, the operational definition of a cluster (≥3 cases within 500 m over 14 days) was locally adapted to the high-density urban context of Bangladesh through consultation with national public health experts. The 14-day temporal window reflects the incubation periods and transmission dynamics of priority syndromes under surveillance, while the 500 m spatial threshold was selected to capture localized transmission patterns in densely populated settings.

### 2.6. Data Collection Procedures

Volunteers conducted household visits four days per week, documenting demographic information, symptoms consistent with case definitions, recent hospitalizations, and any diagnostic tests. GPS coordinates were recorded automatically where possible, otherwise assigned at the ward level.

### 2.7. Digital Platform and Informatics Architecture

Data collection used the Community Health Toolkit (CHT) mobile platform, customized into the “Community Health Tracker” application. Key informatics features included:

• Offline data entry with background encryption
• Automatic queuing and syncing when connectivity became available
• Unique record IDs and timestamps
• Built-in case definitions and conditional logic
• Secure centralized storage on IEDCR servers

Paper forms were used only during connectivity disruptions in the initial phase before the syncing was established and later digitized.

### 2.8. Data Management and Quality Assurance

Daily extraction of records from the central CHT database was performed using Structured Query Language (SQL). Data cleaning and validation were carried out using reproducible Python 3.12.7 scripts within a Jupyter Notebook environment. Key procedures included:

• Deduplication of repeated submissions
• Verification of demographic fields and symptom combinations
• Correction of minor inconsistencies
• GPS validation and ward-level geocoding
• Flagging of anomalous entries for supervisor verification

This ensured an analysis-ready dataset was generated each day.

### 2.9. Data Analysis

Data analysis comprised descriptive epidemiology, spatial analysis, and automated cluster detection. Analysts generated:

• Summaries of age, sex, and comorbidities
• Weekly and monthly trends for each syndrome
• Ward-level maps and heatmaps of suspected cases

### 2.10. Automated Alerts and Public Health Response

When a cluster met the detection criteria, the system generated an automatic alert, accompanied by QGIS-based mapping of affected households. Alerts were disseminated to:

• Public Health Emergency Operations Center (PHEOC)
• IEDCR’s Epidemiology Unit
• Bangladesh Red Crescent Society headquarters

Field response teams evaluated each alert for verification, follow-up visits, or community engagement. These automated alerts represented a critical transformation step within the DIKW informatics model—from raw data to actionable knowledge.

### 2.11. Integration and Data Use

All validated CBS data were integrated into an IEDCR-hosted dashboard [17], enabling real-time visualization of spatial patterns, epidemic curves, and active clusters. Weekly summaries were disseminated to IEDCR units and BDRCS teams.

### 2.12. Data Collection and Real-Time Syncing

The CHT app allowed offline data entry. As CVs worked in the field, they entered records on smartphones; data were automatically encrypted and queued for transmission. When network connectivity was available (usually within 1–2 h), the app synchronized with the central CHT server hosted at IEDCR. Thus, new case reports became available for analysis within hours of collection. Weekly automated reminders were configured to prompt CVs to sync any unsent forms. Supervisors monitored sync status daily; if connectivity issues occurred, paper forms were used temporarily before the syncing was established, with data later entered digitally by CVs.

Each submitted record generated a timestamp and a unique ID. The CHT back-end linked records to an IEDCR central database. No personal identifiers beyond anonymized codes were stored on the national server. The system logged GPS locations, demographics, and symptoms. Real-time data flow ensured that the analytic team could monitor for trends continuously.

### 2.13. Informatics Pipeline: Data Processing and Alerts

Data cleaning and processing were performed continuously. Each morning, the IEDCR data team extracted raw reports via automated SQL queries. In a Jupyter (Python) environment, analysts applied reproducible scripts to clean the data: duplicate records (e.g., due to resubmissions) were identified and removed; inconsistent entries (e.g., spelling errors) were standardized; and geocoding was applied to map each report to the correct ward. Age and gender were checked for validity. Implausible entries (e.g., extreme ages) were flagged and verified. This ensured that the downstream analysis used high-quality data.

Concurrent with cleaning, the analytics pipeline computed weekly summary statistics (new cases per syndrome, per ward) and visualizations. Built-in alert logic scanned the cleaned dataset daily for clusters. When a cluster was detected, the system automatically generated an alert record: it created a QGIS map graphic pinpointing involved households (which is only accessible to the technical team and dealt with considering the ethical issues) with a link to the relevant case details. Alerts were pushed to a shared dashboard and emailed to: (1) the national IEDCR epidemiology team and (2) BDRCS headquarters. This automated alerting provided the “information → knowledge” step in DIKW, highlighting where outbreak signals were emerging in near real time.

### 2.14. Integration and Analytics

All data and alerts were integrated into an IEDCR-hosted analytics portal (using dashboards). Public health officers could view up-to-date maps of syndromic cases, epidemic curves by ward, and active clusters. Weekly reports compiled by the analytic team were shared with district health managers. Plans were made to later import CBS data into the national DHIS2 system for aggregate planning (as is done in some CBS implementations internationally) [18]. Meanwhile, detailed data remained on the national server under IEDCR governance, facilitating cross-checks against IDSR case reports.

## 3. Results

### 3.1. Surveillance Coverage and Activity

From January through June 2025, community volunteers visited 38,489 households and enrolled a total of 128,626 unique individuals, of whom 49.5% were male, 50.4% were female, and 0.1% identified as other. The largest proportion of participants (31.1%) was aged 15–29 years, followed by the 30–39-year age group (18.6%). Children aged 0–4 years accounted for 7.7% of the population, while those aged 5–14 years represented 16.2%. Adults aged 40–49 and 50+ years contributed 13.1% and 13.3%, respectively.

Gender distribution across age groups showed that females outnumbered males in the 15–29 age group (17.2% vs. 13.9%), whereas males were more prevalent in the 40–49 and 50+ age groups. The “Other” category was minimal across all age groups, collectively constituting only 0.1% of the total population (Table 2).

From January to June 2025, the Community-Based Surveillance (CBS) system documented 10,191 alerts and 577 clusters across Dhaka, Rajshahi, and Sylhet (Table 2). Suspected dengue was the most frequently reported syndrome, with 5983 alerts and 334 clusters, predominantly from Rajshahi (61.9% of alerts; 59.0% of clusters), followed by Sylhet (27.8%; 30.5%) and Dhaka (10.3%; 10.5%). Influenza-like illness (ILI) accounted for 1094 alerts and 60 clusters, mainly reported from Dhaka (41.5%; 46.7%) and Sylhet (30.1%; 40.0%). Acute watery diarrhea (AWD) generated 2455 alerts and 151 clusters, largely concentrated in Rajshahi (77.3% of alerts; 85.4% of clusters). Suspected acute jaundice syndrome (AJS) and unusual health events were reported less frequently, with 269 alerts and 7 clusters, and 390 alerts and 25 clusters, respectively, again predominantly from Rajshahi (Table 3).

### 3.2. Spatiotemporal Patterns and Cluster Alerts

Analysis of the cleaned data revealed distinct local outbreak patterns. For instance, suspected dengue case rates surged in specific wards of Rajshahi (Ward 24 saw a sharp peak in April 2025) and in one Dhaka ward (Ward 8) in May. Sylhet city had generally lower overall rates, but Ward 10 experienced a notable dengue spike in May. Seasonal variation was evident: ILI peaked in February in Dhaka wards, while ILI remained low in Rajshahi and Sylhet. AWD signals were highest in Rajshahi (notably spikes in February and April), but nearly absent in Sylhet. Suspected AJS cases were very rare across all cities.

The automated cluster algorithm detected 432 clusters of ≥3 cases across the study period. In Dhaka South, the daily cluster reports were dominated by suspected dengue (29) and ILI (22). AWD contributed sporadic clusters (5) at low levels, while suspected AJS never triggered a cluster in Dhaka. In Rajshahi, suspected dengue clusters were predominant (140); AWD clusters were also consistently reported (89). Sylhet’s clusters were mostly suspected dengue (82) or ILI (20) as well; AWD (12) and suspected AJS (2) clusters were rare. In summary, across all cities, cluster monitoring confirmed suspected dengue and influenza-like illness as the primary outbreak signals. These real-time cluster metrics enabled field teams to prioritize investigations.

### 3.3. Data Quality and Timeliness

The digital pipeline greatly enhanced data quality and timeliness. Over 98% of screened records were synced within 24 h of collection; only 2% required manual upload due to network issues. The automated cleaning scripts caught and resolved >99% of entry errors (e.g., age/date implausibility) without human intervention, compared to baseline estimates of ~10% errors in equivalent paper-based systems. The end result was a continuously updated, high-quality dataset. Weekly analytics (maps, trends) were delivered to district officers within 48 h of the week’s end, compared to previous delays of weeks in paper systems.

No evidence of systematic reporting bias was detected: nearly equal male/female reporting and broad age coverage suggest volunteers captured the full population. A small number of CVs (2 of 36) stopped reporting due to relocation; their wards were covered by CVs from the neighboring areas. During weekly group calls, CVs and supervisors reported that communities were willing to share symptoms (e.g., “community members are no longer afraid to report illness,” according to supervisor feedback).

## 4. Discussion

The Bangladesh CBS pilot demonstrated how public health informatics can be operationalized through community-based surveillance. By embedding digital tools and analytics into the workflow, raw community signals were rapidly converted into actionable intelligence. In DIKW terms, CV interviews produced data (individual symptom reports); data aggregation and cleaning transformed this into information (clean time-stamped, geocoded case logs); spatiotemporal and cluster analyses generated knowledge (insights on outbreak locations and causes); and public health teams applied this wisdom via targeted investigations. As Jung et al. noted from Somaliland, engaging community volunteers and digitizing their reports can indeed prevent potential outbreaks [19]. Our findings corroborate that approach: signals like suspected dengue hotspots were detected days earlier than facility surveillance might have.

Several aspects of informatics are key. First, mobile data collection ensured timely and accurate capture of health events. Using the CHT app meant all data were standardized upon entry, immediately geotagged, and available for syncing. Studies have shown that electronic data collection greatly reduces missing or inconsistent data compared to paper forms [20]. Although we did not formally compare to paper, in practice, the app eliminated hours of manual data entry and associated errors. Other CBS programs have similarly reported high fidelity of volunteer reports when using digital tools. For example, the Somaliland study found that 83–97% of community signals matched case definitions and were escalated—a very high rate of valid alerts [19]. In Bangladesh, our digital checklists (with required fields) likewise kept completeness high; invalid entries were below 1% and quickly caught by automated filters.

Second, real-time syncing was crucial. Because CVs synced data as soon as they returned to internet coverage areas, the central server always had near-current data. This contrasts with traditional reporting that can lag weeks. In Ghana’s COVID-19 response, linking CBS with real-time GIS mapping was credited with a four-fold jump in case detection. We saw a similar phenomenon: once the pilot began, CBS reported many cases (especially sporadic suspected dengue) that had been going unreported.

**Figure 1:**
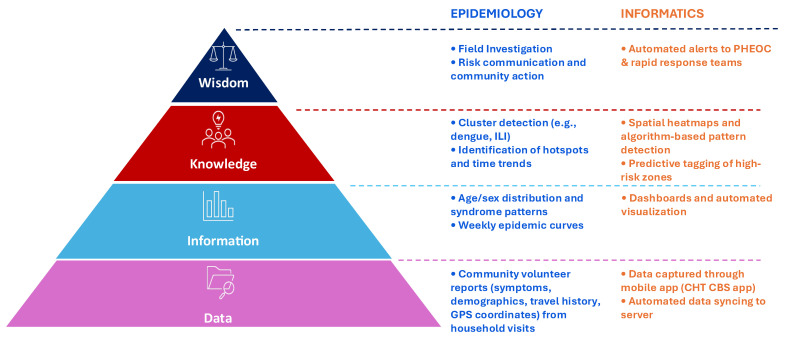
DIKW Pyramid showing how epidemiology and public health informatics blended through Community-Based Surveillance in Bangladesh.

Third, automated alerts and analytics turned data into active responses. The cluster-detection algorithm is an example of simple “event-based surveillance” logic embedded in software. Without it, team members might overlook gradually rising case counts in noisy data. Instead, daily alerts (sent with a map) highlighted exactly where to focus. This practice aligned with WHO guidance on CBS as an early warning: community reporters can “alert health authorities to respond in a timely manner” when unusual symptom clusters appear [21]. Indeed, our analytic pipeline (SQL extraction → Python cleaning → GIS mapping) functions like a real-time epidemiological surveillance system. Each step adds value: cleaning ensures information accuracy, clustering analysis yields knowledge of emerging threats, and alerts deliver wisdom to health workers on action priorities (Figure 1).

This pattern is consistent with findings from CBS implementations in Sierra Leone, Ghana, and Indonesia, where community-level reporting enhanced early detection of outbreak signals and complemented routine surveillance systems [9,10,11]. In Ghana, augmenting COVID-19 surveillance with CBS and GIS contact tracing significantly raised the case discovery rate [10]. Although that project was for COVID-19, it underlines a general principle: adding community data sources makes surveillance more sensitive. In our CBS, suspected dengue and influenza outbreaks were detected at the community level before their reporting through health facilities. Similarly, Mergenthaler et al. in Sierra Leone found that CBS alerts “mirrored and added to” facility reports, indicating CBS caught cases the formal system did not [21].

Technically, integrating CBS data into national health systems is the next step. Globally, countries are moving to incorporate CBS into platforms like DHIS2. For instance, the Lebanese Red Cross now feeds CBS reports directly into the Ministry of Health’s DHIS2 dashboard, enabling early outbreak spotting [18]. Although we did not fully link our pilot to Bangladesh’s DHIS2, the CHT database was designed to match DHIS2 data models, facilitating future import. As Wallis et al. (2023) observed, tools like DHIS2 Tracker and Community Health Toolkit can fill all necessary attributes for CBS workflows [20]. In Bangladesh’s context, integrating with DHIS2 (the system already used for disease reporting from government health facilities) would make community data part of the regular surveillance flow, overcoming one challenge of sustainability.

Another insight relates to the DIKW learning loop. By reviewing weekly analytics with local teams, we turned knowledge back into action. For example, once a suspected dengue cluster was confirmed, teams engaged community members to remove mosquito breeding sites and distributed information on early care-seeking. This reflects the “wisdom” end of the DIKW pyramid: community-data-informed interventions. Training materials for CBS increasingly emphasize this feedback loop—not just detecting an outbreak but closing the loop with an informed response. Globally, community volunteers have been leveraged for action (e.g., 7-1-7 initiative) [22], and CBS follows that model.

This study was conducted in purposively selected urban wards, which may limit generalizability to rural or hard-to-reach settings where population density, healthcare access, and reporting dynamics differ. Urban environments may also facilitate cluster detection due to higher population density and mobility; therefore, findings should be interpreted within this context. The current analysis was primarily descriptive, reflecting the early implementation phase of the CBS system and focusing on feasibility and operational performance. While this approach demonstrates the system’s functionality, more advanced analyses—such as regression modeling, spatial risk mapping, and identification of demographic or environmental determinants—are needed to strengthen epidemiological insights and are planned for subsequent phases using expanded datasets. In addition, formal evaluation of surveillance performance, including sensitivity, positive predictive value, and comparison with facility-based systems, was beyond the scope of this study. Nevertheless, proxy indicators such as high data synchronization rates (>98% within 24 h) suggest strong system timeliness. Additionally, variation in volunteer training, reporting fatigue, and potential misclassification of syndromic conditions may have influenced reporting accuracy. Finally, reliance on community volunteer–based reporting may introduce reporting bias, and some degree of underreporting or differential reporting cannot be entirely excluded.

## 5. Conclusions

Community-based surveillance, strengthened through digital tools and real-time analytics, proved to be a powerful public health informatics strategy for early outbreak detection and response in Bangladesh. During the pilot period, the system monitored 128,626 individuals across 38,489 households and generated 10,191 alerts and 577 clusters, demonstrating its capacity to capture large-scale, real-time community health signals. By enabling community volunteers to report digitally and linking those reports to automated cleaning, cluster detection, and GIS mapping, the system effectively operationalized the DIKW continuum—transforming raw data into actionable intelligence. This translation supported concrete public health actions, including targeted outbreak investigations, community engagement, and localized vector control measures in identified hotspot areas. However, the findings should be interpreted in light of the urban-only implementation, which may limit generalizability to rural settings with different transmission dynamics and health system access. Integrating CBS with national platforms such as DHIS2 will further amplify its value while ensuring long-term sustainability, interoperability, and alignment with national surveillance systems.

## Figures and Tables

**Table 1:** Community Case Definitions Used in the CBS Piloting in Bangladesh.

Syndrome	Community Case Definition
Suspected Dengue	A patient with onset of acute fever (more than or equal to 100 °F) within 10 days and at least one of these symptoms—rash, headache, myalgia, arthralgia/arthritis, conjunctivitis, hemorrhagic manifestations, or retro-orbital pain.
Influenza-like Illness (ILI)	An acute respiratory infection with a measured fever of ≥38 °C and cough with onset within the last 10 days.
Acute Watery Diarrhea (AWD)	Three or more loose or liquid stools over a period of 24 h
Suspected Acute Jaundice Syndrome (AJS)	Any person with fever and yellowish discoloration in the white part of the eyes or yellowing of the skin within two weeks of the onset of the first symptoms.
Unusual health events	If three or more people suddenly fall ill or die with similar unknown signs or symptoms within a 1 km radius or an area equivalent to a 10 min walking distance in the past 2 weeks.

**Table 2:** Gender and age group distribution of participants of CBS from Jan to Jun 2025.

Age Groups (Years)	Male *n* (%)	Female *n* (%)	Other *n* (%)	Total *n* (%)
0–4	4844 (3.8)	4971 (3.9)	78 (0.1)	9893 (7.7)
5–14	10,605 (8.2)	10,271 (8.0)	2 (0.0)	20,878 (16.2)
15–29	17,932 (13.9)	22,075 (17.2)	10 (0.0)	40,017 (31.1)
30–39	11,882 (9.2)	12,067 (9.4)	2 (0.0)	23,951 (18.6)
40–49	9149 (7.1)	7684 (6.0)	2 (0.0)	16,835 (13.1)
50+	9270 (7.2)	7780 (6.0)	2 (0.0)	17,052 (13.3)
Total	63,682 (49.5)	64,848 (50.4)	96 (0.1)	128,626 (100.0)

**Table 3:** District-wise distribution of reports of alerts and clusters of different syndromes of CBS from Jan–Jun 2025.

District	Suspected Dengue		ILI		AWD		Suspected AJS		Unusual Health Event		Total	
	Alert *n* (%)	Cluster *n* (%)	Alert *n* (%)	Cluster *n* (%)	Alert *n* (%)	Cluster *n* (%)	Alert *n* (%)	Cluster *n* (%)	Alert *n* (%)	Cluster *n* (%)	Alert *n* (%)	Cluster *n* (%)
Dhaka	615 (10.3)	35 (10.5)	454 (41.5)	28 (46.7)	214 (8.7)	6 (4.0)	39 (14.5)	0 (0.0)	36 (9.2)	6 (24.0)	1358 (13.3)	75 (13.0)
Rajshahi	3704 (61.9)	197 (59.0)	311 (28.4)	8 (13.3)	1898 (77.3)	129 (85.4)	137 (50.9)	5 (71.4)	311 (79.7)	8 (32.0)	6361 (62.4)	347 (60.1)
Sylhet	1664 (27.8)	102 (30.5)	329 (30.1)	24 (40.0)	343 (14.0)	16 (10.6)	93 (34.6)	2 (28.6)	43 (11.0)	11 (44.0)	2472 (24.3)	155 (26.9)
Total	5983 (100)	334 (100)	1094 (100)	60 (100)	2455 (100)	151 (100)	269 (100)	7 (100)	390 (100)	25 (100)	10,191 (100)	577 (100)

## Data Availability

The data presented in this study are available on reasonable request from the corresponding author. The data are not publicly available due to ethical and confidentiality restrictions, as they contain sensitive health and geolocation information collected through a national surveillance system. Access to the data is subject to approval by the Institute of Epidemiology, Disease Control and Research (IEDCR), Bangladesh.
